# Comparison of Different Convolutional Neural Network Activation Functions and Methods for Building Ensembles for Small to Midsize Medical Data Sets

**DOI:** 10.3390/s22166129

**Published:** 2022-08-16

**Authors:** Loris Nanni, Sheryl Brahnam, Michelangelo Paci, Stefano Ghidoni

**Affiliations:** 1 Department of Information Engineering, University of Padua, Via Gradenigo 6, 35131 Padova, Italy; 2 Department of Information Technology and Cybersecurity, Missouri State University, 901 S. National Street, Springfield, MO 65804, USA; 3 BioMediTech, Faculty of Medicine and Health Technology, Tampere University, Arvo Ylpön katu 34, D 219, FI-33520 Tampere, Finland

**Keywords:** convolutional neural networks, activation functions, biomedical classification, ensembles, MeLU variants

## Abstract

CNNs and other deep learners are now state-of-the-art in medical imaging research. However, the small sample size of many medical data sets dampens performance and results in overfitting. In some medical areas, it is simply too labor-intensive and expensive to amass images numbering in the hundreds of thousands. Building Deep CNN ensembles of pre-trained CNNs is one powerful method for overcoming this problem. Ensembles combine the outputs of multiple classifiers to improve performance. This method relies on the introduction of diversity, which can be introduced on many levels in the classification workflow. A recent ensembling method that has shown promise is to vary the activation functions in a set of CNNs or within different layers of a single CNN. This study aims to examine the performance of both methods using a large set of twenty activations functions, six of which are presented here for the first time: 2D Mexican ReLU, TanELU, MeLU + GaLU, Symmetric MeLU, Symmetric GaLU, and Flexible MeLU. The proposed method was tested on fifteen medical data sets representing various classification tasks. The best performing ensemble combined two well-known CNNs (VGG16 and ResNet50) whose standard ReLU activation layers were randomly replaced with another. Results demonstrate the superiority in performance of this approach.

## 1. Introduction

First developed in the 1940s, artificial neural networks have had a checkered history, sometimes lauded by researchers for their unique computational powers and other times discounted for being no better than statistical methods. About a decade ago, the power of deep artificial neural networks radically changed the direction of machine learning and rapidly made significant inroads into many scientific, medical, and engineering areas [1,2,3,4,5,6,7,8]. The strength of deep learners is demonstrated by the many successes achieved by one of the most famous and robust deep learning architectures, Convolutional Neural Networks (CNNs). CNNs frequently win image recognition competitions and have consistently outperformed other classifiers on a variety of applications, including image classification [9,10], object detection [11,12], face recognition [13,14], and machine translation [15], to name a few. Not only do CNNs continue to perform better than traditional classifiers, but they also outperform human beings, including experts, in many image recognition tasks. In the medical domain, for example, CNNs have been shown to outperform human experts in recognizing skin cancer [16], skin lesions on the face and scalp [17], and the detection of esophageal cancer [18].

It is no wonder, then, that CNNs and other deep learners have exploded exponentially in medical imaging research [19]. CNNs have been successfully applied to a wide range of applications (as evidenced by these very recent reviews and studies): the identification and recognition of facial phenotypes of genetic disorders [20], diabetic retinopathy [21,22,23], glaucoma [24], lung cancer [25], breast cancer [26], colon cancer [27], gastric cancer [28,29], ovarian cancer [30,31,32], Alzheimer’s disease [33,34], skin cancer [16], skin lesions [17], oral cancer [35,36], esophageal cancer [18], and GI ulcers [37].

Despite these successes, the unique characteristics of medical images pose challenges for CNN classification. The first challenge concerns the image size of medical data. Typical CNN image inputs are around 200 × 200 pixels. Many medical images are gigantic. For instance, histopathology slides, once digitized, often result in gigapixel images, around 100,000 × 100,000 pixels [38]. Another problem for CNNs is the small sample size of many medical data sets. As is well known, CNNs require massive numbers of samples to prevent overfitting. It is too cumbersome, labor-intensive, and expensive to acquire collections of images numbering in the hundreds of thousands in some medical areas. There are well-known techniques for tackling the problem of overfitting when data are low, the two most common being transfer learning with pre-trained CNNs and data argumentation. The medical literature is replete with studies using both methods (for some literature reviews on these two methods in medical imaging, see [39,40,41]). As observed in [40], transfer learning works well combined with data augmentation. Transfer learning is typically applied in two ways: for fine-tuning with pre-trained CNNs and as feature extractors, with the features then fed into more traditional classifiers.

Building Deep CNN ensembles of pre-trained CNNs is yet another powerful technique for enhancing CNN performance on small sample sizes. Some examples of robust CNN ensembles reported in the last couple of years include [42], for classifying ER status from DCE-MRI breast volumes; [43], where a hierarchical ensemble was trained for diabetic macular edema diagnosis; [44] for whole-brain segmentation; and [45] for small lesion detection.

Ensemble learning combines outputs from multiple classifiers to improve performance. This method relies on the introduction of diversity, whether in the data each CNN is trained on, the type of CNNs used to build the ensemble, or some other changes in the architecture of the CNNs. For example, in [43], mentioned above, ensembles were built on the classifier level by combining the results of two sets of CNNs within a hierarchical schema. In [44], a novel ensemble was developed on the data level by looking at different brain areas, and in [45], multiple-depth CNNs were trained on image patches. In [46], CNNs with different activation functions were shown to be highly effective, and in [47], different activation functions were inserted into different layers within a single CNN network.

In this paper, we extend [46] by testing several activation functions with two CNNs, VGG16 [48] and ResNet50 [49], and their fusions across fifteen biomedical data sets representing different biomedical classification tasks. The set of activation functions includes the best-performing ones used with these networks and six new ones: 2D Mexican ReLU, TanELU, MeLU + GaLU, Symmetric MeLU, Symmetric GaLU, and Flexible MeLU. The best performance was obtained by randomly replacing every ReLU layer of each CNN with a different activation function.

The contributions of this study are the following:

(1) The performance of twenty individual activation functions is assessed using two CNNs (VGG16 and ResNet50) across fifteen different medical data sets.
(2) The performance of ensembles composed of the CNNs examined in #1 and four other topologies is evaluated.
(3) Six new activation functions are proposed.

The remainder of this paper is organized as follows. In Section 2, we review the literature on activation functions used with CNNs. In Section 3, we describe all the activation functions tested in this work. In Section 4, the stochastic approach for constructing CNN ensembles is detailed (some other methods are described in the experimental section). In Section 5, we present a detailed evaluation of each of the activation functions using both CNNs on the fifteen data sets, along with the results of their fusions. Finally, in Section 6, we suggest new ideas for future investigation together with some concluding remarks. 

The MATLAB source code for this study will be available at https://github.com/LorisNanni.

## 2. Related Work with Activation Functions

Evolutions in CNN design initially focused on building better network topologies. As activation functions impact training dynamics and performance, many researchers have also focused on developing better activation functions. For many years, the sigmoid and the hyperbolic tangent were the most popular neural network activation functions. The hyperbolic tangent’s main advantage over the sigmoid is that the hyperbolic has a steeper derivative than the sigmoid function. Neither function, however, works that well with deep learners since both are subject to the vanishing gradient problem. It was soon realized that nonlinearities work better with deep learners.

One of the first nonlinearities to demonstrate improved performance with CNNs was the Rectified Linear Unit (ReLU) activation function [50], which is equal to the identity function with positive input and zero with negative input [51]. Although ReLU is nondifferentiable, it gave AlexNet the edge to win the 2012 ImageNet competition [52].

The success of ReLU in AlexNet motivated researchers to investigate other nonlinearities and the desirable properties they possess. As a consequence, variations of ReLU have proliferated. For example, Leaky ReLU [53], like ReLU, is also equivalent to the identity function for positive values but has a hyperparameter α  > 0 applied to the negative inputs to ensure the gradient is never zero. As a result, Leaky ReLU is not as prone to getting caught in local minima and solves the ReLU problem with hard zeros that makes it more likely to fail to activate. The Exponential Linear Unit (ELU) [54] is an activation function similar to Leaky ReLU. The advantage offered by ELU is that it always produces a positive gradient since it exponentially decreases to the limit point α as the input goes to minus infinity. The main disadvantage of ELU is that it saturates on the left side. Another activation function designed to handle the vanishing gradient problem is the Scaled Exponential Linear Unit (SELU) [55]. SELU is identical to ELU except that it is multiplied by the constant λ>1 to maintain the mean and the variance of the input features.

Until 2015, activation functions were engineered to modify the weights and biases of a neural network. Parametric ReLU (PReLU) [56] gave Leaky ReLU a learnable parameter applied to the negative slope. The success of PReLU attracted more research on the learnable activation functions topic [57,58]. A new generation of activation functions was then developed, one notable example being the Adaptive Piecewise Linear Unit (APLU) [57]. APLU independently learns during the training phase the piecewise slopes and points of nondifferentiability for each neuron using gradient descent; therefore, it can imitate any piecewise linear function.

Instead of employing a learnable parameter in the definition of an activation function, as with PReLu and APLU, the construction of an activation function from a given set of functions can be learned. In [59], for instance, it was proposed to create an activation function that automatically learned the best combinations of tanh, ReLU, and the identity function. Another activation function of this type is the S-shaped Rectified Linear Activation Unit (SReLU) [60]. Using reinforcement learning, SReLU was designed to learn convex and nonconvex functions to imitate both the Webner–Fechner and the Stevens law. This process produced an activation called Swish, which the authors view as a smooth function that nonlinearly interpolates between the linear function and ReLU.

Similar to APLU is the Mexican ReLU (MeLU [61]), whose shape resembles the Mexican hat wavelet. MeLU is a piecewise linear activation function that combines PReLU with many Mexican hat functions. Like APLU, MeLU has learnable parameters that approximate the same piecewise linear functions equivalent to identity when x is sufficiently large. MeLU has some main differences with respect to APLU: first, it has a much larger number of parameters; and second, the method in which the approximations are calculated for each function is different.

## 3. Activation Functions

As described in the Introduction, this paper explores classifying medical imagery using combinations of some of the best performing activation functions on two widely used high-performance CNN architectures: VGG16 [48] and ResNet50 [49], each pre-trained on ImageNet. VGG16 [48], also known as the OxfordNet, is the second-place winner in the ILSVRC 2014 competition and was one of the deepest neural networks produced at that time. The input into VGG16 passes through stacks of convolutional layers, with filters having small receptive fields. Stacking these layers is similar in effect to CNNs having larger convolutional filters, but the stacks involve fewer parameters and are thus more efficient. ResNet50 [49], winner of the ILSVRC 2015 contest and now a popular network, is a CNN with fifty layers known for its skip connections that sum the input of a block to its output, a technique that promotes gradient propagation and that propagates lower-level information to higher level layers.

The remainder of this section mathematically describes and discusses the twenty activation functions investigated in this study: ReLU [50], Leaky ReLU [62], ELU [54], SELU [55], PReLU [56], APLU [57], SReLU [63], MeLU [61], Splash [64], Mish [65], PDELU [66], Swish [60], Soft Learnable [67], SRS [67], and GaLU ([68]), as well as the novel activation functions proposed here: 2D Mexican ReLU; TanELU; MeLU + GaLU; Symmetric MeLU; Symmetric GaLU; Flexible MeLU.

The main advantage of these more complex activation functions with learnable parameters is that they can better learn the abstract features through nonlinear transformations. This is a generic characteristic of learnable activation functions, well known in shallow networks [69]. The main disadvantage is that activation functions with several learnable parameters need large data sets for training.

A further rationale for our proposed activation functions is to create activation functions that are quite different from each other to improve performance in ensembles; for this reason, we have developed the 2D MeLU, which is quite different from standard activation functions.

### 3.1. Rectified Activation Functions

#### 3.1.1. ReLU

**Figure 1:**
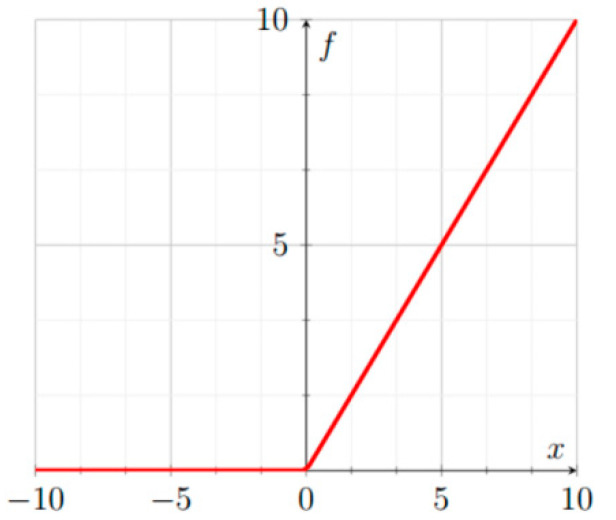
ReLU.

ReLU [50], illustrated in Figure 1, is defined as:

(1) $$y_{i}=f\left(x_{i}\right)=\left\{\begin{array}{ccc} 0, & | & x_{i}<0\\ x_{i}, & | & x_{i}\geq 0. \end{array}\right.$$

The gradient of ReLU is

(2) $$\frac{dy_{i}}{dx_{i}}=f\prime \left(x_{i}\right)=\left\{\begin{array}{ccc} 0, & | & x_{i}<0\\ 1, & | & x_{i}\geq 0. \end{array}\;\;\;\;\;\;\;\;\;\;\;\;\;\;\;\;\;\;\right.$$

#### 3.1.2. Leaky ReLU

**Figure 2:**
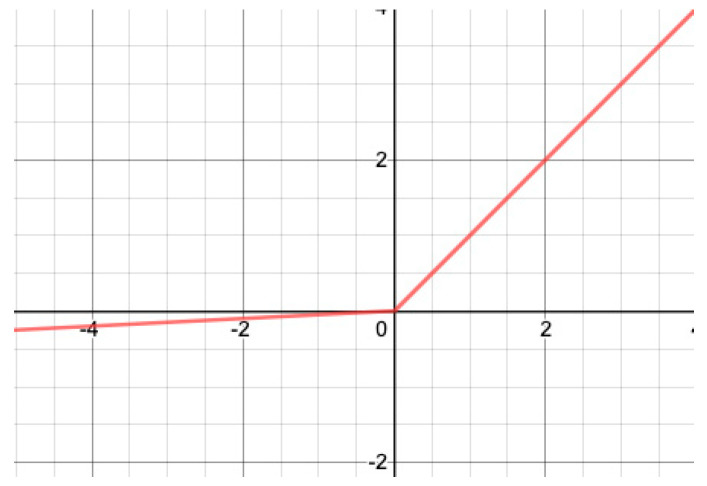
Leaky ReLU.

In contrast to ReLU, Leaky ReLU [53] has no point with a null gradient. Leaky ReLU, illustrated in Figure 2, is defined as:

(3) $$y_{i}=f\left(x_{i}\right)=\left\{\begin{array}{ccc} ax_{i}, & | & x_{i}<0\\ \;\;\;x_{i}, & | & x_{i}\geq 0, \end{array}\right.$$

where a (set to 0.01 here) is a small real number. 

The gradient of Leaky ReLU is:

(4) $$\frac{dy_{i}}{dx_{i}}=f^{\prime \left(x_{i}\right)}=\left\{\begin{array}{ccc} a, & | & x_{i}<0\\ 1, & | & x_{i}\geq 0. \end{array}\right.$$

#### 3.1.3. PReLU

Parametric ReLU (PreLU) [56] is identical to Leaky ReLU except that the parameter a_c_ (different for every channel of the input) is learnable. PReLU is defined as:

(5) $$y_{i}=f\left(x_{i}\right)=\left\{\begin{array}{ccc} a_{c}x_{i}, & | & x_{i}<0\\ \;\;\;x_{i}, & | & x_{i}\geq 0, \end{array}\right.$$

where $a_{c}$ is a real number.

The gradients of PReLU are:

(6) $$\frac{dy_{i}}{dx_{i}}=f^{\prime \left(x_{i}\right)}=\left\{\begin{array}{ccc} a_{c}, & | & x_{i}<0\\ \;1, & | & x_{i}\geq 0, \end{array}\right.$$

(7) $$\frac{dy_{i}}{da_{c}}=\left\{\begin{array}{ccc} x_{i}, & | & x_{i}<0\\ \;0, & | & x_{i}\geq 0. \end{array}\right.$$

Slopes on the left-hand sides are all initialized to 0.

### 3.2. Exponential Activation Functions

#### 3.2.1. ELU

**Figure 3:**
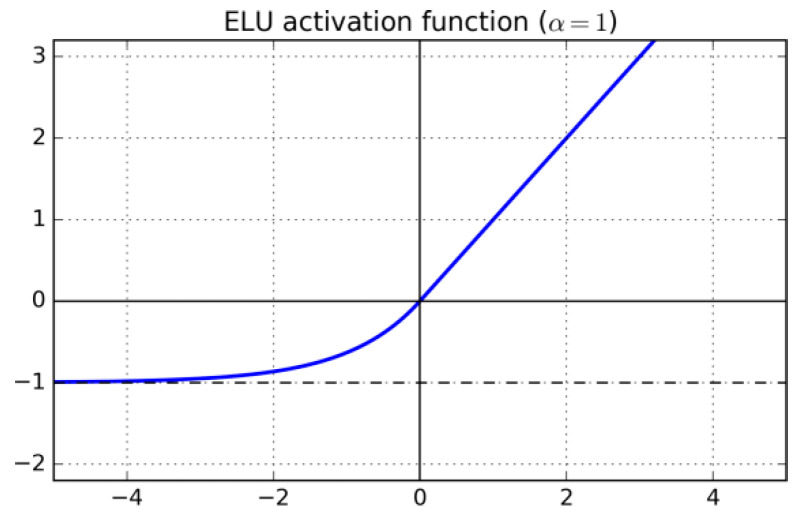
ELU.

Exponential Linear Unit (ELU) [54] is differentiable and, as is the case with Leaky ReLU, the gradient is always positive and bounded from below by −a. ELU, illustrated in Figure 3, is defined as:

(8) $$y_{i}=f\left(x_{i}\right)=\left\{\begin{array}{ccc} a\left(\exp(x_{i})-1\right), & | & x_{i}<0\\ x_{i}, & | & x_{i}\geq 0, \end{array}\right.$$

where a (set to 1 here) is a real number. 

The gradient of Leaky ELU is:

(9) $$\frac{dy_{i}}{dx_{i}}=f^{\prime \left(x_{i}\right)}=\left\{\begin{array}{ccc} a\;exp\left(x_{i}\right), & | & x_{i}<0\\ 1, & | & x_{i}\geq 0. \end{array}\right.$$

#### 3.2.2. PDELU

Piecewise linear Parametric Deformable Exponential Linear Unit (PDELU) [66] is designed to have zero mean, which speeds up the training process. PDELU is defined as

(10) $$y_{i}=f\left(x_{i}\right)=\left\{\begin{array}{cc} x_{i} & x_{i}>0\\ \alpha_{i}\cdot \left({\left[1+\left(1-t\right)x_{i}\right]}_{}^{\frac{1}{1-t}}-1\right) & x_{i}\leq 0 \end{array}\right.$$

where ${\left[x\right]}_{+}=\max\left(x,0\right)$. The $f\left(x_{i}\right)$ function takes values in the $\left(-\alpha ,\infty \right)$ range; its slope in the negative part is controlled by means of the $\alpha_{i}$ parameters (i runs over the input channels) that are jointly learned by the loss function. The parameter t controls the degree of deformation of the exponential function. If 0<t<1, then $f\left(x_{i}\right)$ decays to 0 faster than the exponential.

### 3.3. Logistic Sigmoid and Tanh-Based AFs

#### 3.3.1. Swish

Swish [60] is designed using reinforcement learning to learn to efficiently sum, multiply, and compose different functions that are used as building blocks. The best function is

(11) $$y=f\left(x\right)=x\cdot sigmoid\left(\beta x\right)=\frac{x}{1+e^{-\beta x}}$$

where β acts as a constant or a learnable parameter that is evaluated during training. When β=1, as in this study, Swish is equivalent to the Sigmoid-weighted Linear Unit (SiLU), proposed for reinforcement learning. As β→∞, Swish assumes the shape of a ReLU function. Unlike ReLU, however, Swish is smooth and nonmonotonic, as demonstrated in [60]; this is a peculiar aspect of this activation function. In practice, a value of β=1 is a good starting point, from which performance can be further improved by training such a parameter.

#### 3.3.2. Mish

Mish [65] is defined as

(12) $$y=f\left(x\right)=x\cdot tanh\left(softplus\left(\alpha x\right)\right)=x\cdot tanh\left(ln\left(1+e^{\alpha x}\right)\right),$$

where α is a learnable parameter.

#### 3.3.3. TanELU (New)

TanELU is an activation function presented here that is simply the weighted sum of tanh and ReLU:

(13) $$y_{i}=ReLU\left(x_{i}\right)+a_{i}tanh\left(x_{i}\right),\;$$

where $a_{i}$ is a learnable parameter.

### 3.4. Learning/Adaptive Activation Functions

#### 3.4.1. SReLU

**Figure 4:**
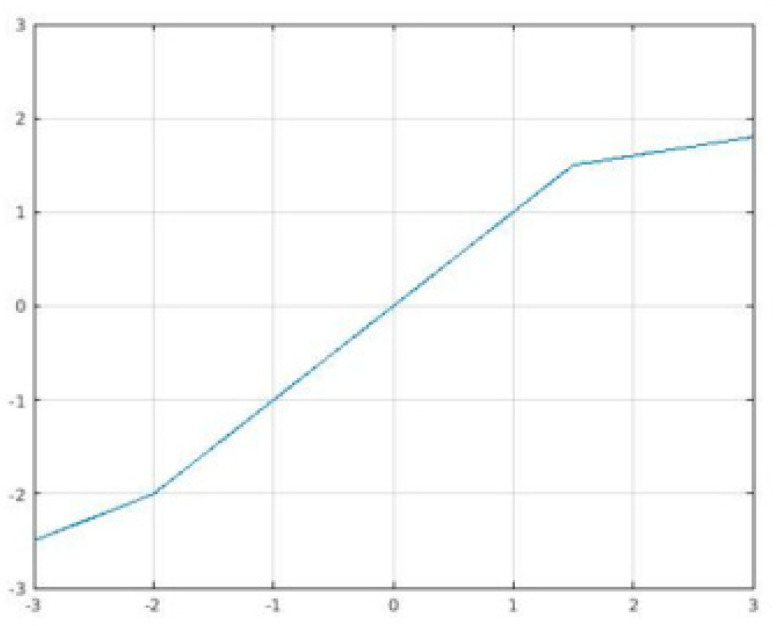
SReLU.

S-shaped ReLU (SReLU) [63] is composed of three piecewise linear functions expressed by four learnable parameters ($t^{l},t^{r},a^{l}$, and $a^{r}$ initialized as $a^{l}=0,\;t^{l}=0$, $t^{r}=maxInput$, a hyperparameter). This rather large set of parameters gives SReLU its high representational power. SReLU, illustrated in Figure 4, is defined as:

(14) $$y_{i}=f\left(x_{i}\right)=\left\{\begin{array}{ccc} t^{l}+a^{l}\left(x_{i}-t^{l}\right), & | & x_{i}\leq t^{l}\\ x_{i}, & | & t^{l}<x_{i}<t^{r}\\ t^{r}+a^{r}\left(x_{i}-t^{r}\right), & | & x_{i}\geq t^{r}. \end{array}\right.,\;$$

where $a_{c}$ is a real number. 

The gradients of SeLU are:

(15) $$\frac{dy_{i}}{dx_{i}}=f\prime \left(x_{i}\right)=\left\{\begin{array}{ccc} a^{l}, & | & x_{i}\leq t^{l}\\ 1, & | & t^{l}<x_{i}<t^{r}\\ a^{r}, & | & x_{i}\geq t^{r} \end{array}\right.,$$

(16) $$\frac{dy_{i}}{da^{l}}=\left\{\begin{array}{ccc} x_{i}-t^{l}, & | & x_{i}\leq t^{l}\\ 0, & | & x_{i}>t^{l}, \end{array}\right.$$

(17) $$\frac{dy_{i}}{dt^{l}}=\left\{\begin{array}{ccc} 1-a^{l}, & | & x_{i}\leq t^{l}\\ 0, & | & x_{i}>t^{l}, \end{array}\right.$$

(18) $$\frac{dy_{i}}{da^{r}}=\left\{\begin{array}{ccc} x_{i}-t^{r}, & | & x_{i}\geq t^{r}\\ 0, & | & x_{i}<t^{r}, \end{array}\right.$$

(19) $$\frac{dy_{i}}{dt^{r}}=\left\{\begin{array}{ccc} 1-a^{r}, & | & x_{i}\geq t^{r}\\ 0, & | & x_{i}<t^{r}. \end{array}\right.$$

Here, we use $a^{l}=0.5,a^{r}=0.2,t^{l}=-2,t^{r}=1.5.$

#### 3.4.2. APLU

Adaptive Piecewise Linear Unit (APLU) [57] is a linear piecewise function that can approximate any continuous function on a compact set. The gradient of APLU is the sum of the gradients of ReLU and of the functions contained in the sum. APLU is defined as:

(20) $$y_{i}=\;\mathrm{ReLU}\left(x_{i}\right)+\sum_{c=1}^{n}a_{c}\min\left(0,-x_{i}+b_{c}\right),$$

where $a_{c}$ and $b_{c}$ are real numbers that are different for each channel of the input.

With respect to the parameters $a_{c}$ and $b_{c}$, the gradients of APLU are:

(21) $$\frac{df\left(x,a\right)}{da_{c}}=\left\{\begin{array}{ccc} -x+b_{c}, & | & x<b_{c}\\ 0, & | & x\geq b_{c} \end{array}\right.,$$

(22) $$\frac{df\left(x,a\right)}{db_{c}}=\left\{\begin{array}{ccc} a_{c}, & | & x<b_{c}\\ 0, & | & x\geq b_{c}. \end{array}\right.$$

The values for $a_{c}$ are initialized here to zero, with points randomly initialized. The 0.001 $L^{2}$-penalty is added to the norm of the $a_{c}$ values. This addition requires that another term $L^{reg}$ be included in the loss function:

(23) $$L^{\mathrm{reg}}=\sum_{c=1}^{n}{\left|a_{c}\right|}^{2}.$$

Furthermore, a relative learning rate is added: maxInput multiplied by the smallest value used for the rest of the network. If λ is the global learning rate, then the learning rate $\lambda^{*}$ of the parameters $a_{c}$ would be

(24) $$\lambda^{*}=\lambda /maxInput.$$

#### 3.4.3. MeLU

The mathematical basis of the Mexican ReLU (MeLU) [61] activation function can be described as follows. Given the real numbers a and λ and letting $\phi^{a,\;\lambda }\left(x\right)=\max\left(\lambda -\left|x-a\right|,0\right)$ be a so-called Mexican hat type of function, then when $\left|x-a\right|>\lambda$, the function $\phi^{a,\;\lambda }\left(x\right)$ is null but increases with a derivative of 1 and a between a−λ and decreases with a derivative of −1 between a and a+λ.

Considering the above, MeLU is defined as

(25) $$y_{i}=MeLU\left(x_{i}\right)=PReLU^{c_{0}}\left(x_{i}\right)+\sum_{j=1}^{k-1}c_{j}\;\phi^{a_{j},\lambda_{j}}\left(x_{i}\right),\;$$

where k is the number of learnable parameters for each channel, $c_{j}$ are the learnable parameters, and $c_{0}$ is the vector of parameters in PReLU.

The parameter k (k=4 or 8 here) has one value for PReLU and k−1 values for the coefficients in the sum of the Mexican hat functions. The real numbers $a_{j}$ and $\lambda_{j}$ are fixed (see Table 1) and are chosen recursively. The value of maxInput is set to 256. The first Mexican hat function has its maximum at 2·maxInput and is equal to zero in 0 and 4·maxInput. The next two functions are chosen to be zero outside the interval [0, 2·maxInput] and [2·maxInput, 4·maxInput], with the requirement being they have their maximum in maxInput and 3·maxInput.

The Mexican hat functions on which MeLU is based are continuous and piecewise differentiable. Mexican hat functions are also a Hilbert basis on a compact set with the $L^{2}$ norm. As a result, MeLU can approximate every function in $L^{2}\left(\left[0,\;1024\right]\right)$ as k goes to infinity.

When the $c_{i}$ learnable parameters are set to zero, MeLU is identical to ReLU. Thus, MeLU can easily replace networks pre-trained with ReLU. This is not to say, of course, that MeLU cannot replace the activation functions of networks trained with Leaky ReLU and PReLU. In this study, all $c_{i}$ are initialized to zero, so start off as ReLU, with all its attendant properties.

MeLU’s hyperparameter ranges from zero to infinity, producing many desirable properties. The gradient is rarely flat, and saturation does not occur in any direction. As the size of the hyperparameter approaches infinity, it can approximate every continuous function on a compact set. Finally, the modification of any given parameter only changes the activation on a small interval and only when needed, making optimization relatively simple.

#### 3.4.4. GaLU

Piecewise linear odd functions, composed of many linear pieces, do a better job in approximating nonlinear functions compared to ReLU [70]. For this reason, Gaussian ReLU (GaLU) [68], based on Gaussian types of functions, aims to add more linear pieces with respect to MeLU. Since GaLU extends MeLU, GaLU retains all the favorable properties discussed in Section 3.4.3.

Letting $\phi_{g}{}^{a,\;\lambda }\left(x\right)=\max\left(\lambda -\left|x-a\right|,0\right)$+min ($\left|x-a-2\lambda \right|-\lambda ,0$) be a Gaussian type of function, where a and λ are real numbers, GaLU is defined, similarly to MeLU, as

(26) $$y_{i}=GaLU\left(x_{i}\right)=PReLU^{c_{0}}\left(x_{i}\right)+\sum_{j=1}^{k-1}c_{j}\;\phi_{g}{}^{a_{j},\lambda_{j}}\left(x_{i}\right).$$

In this work, k=2 parameters for what will be called in the experimental section Small GaLU and k=4 for GaLU proper.

Like MeLU, GaLU has the same set of fixed parameters. A comparison of values for the fixed parameters with maxInput=1 is provided in Table 2.

#### 3.4.5. SRS

Soft Root Sign (SRS) [67] is defined as

(27) $$y=f\left(x\right)=\frac{x}{\frac{x}{\alpha }+e^{-\frac{x}{\beta }}},$$

where α and β are nonnegative learnable parameters. The output has zero means if the input is a standard normal.

#### 3.4.6. Soft Learnable

It is defined as

(28) $$y=f\left(x\right)=\left\{\begin{array}{ccc} x, & | & x>0\\ \alpha \cdot ln\left(\frac{1+e^{\beta x}}{2}\right), & | & x\leq 0, \end{array}\right.$$

where α and β are nonnegative trainable parameters that enable SRS to adaptively adjust its output to provide a zero-mean property for enhanced generalization and training speed. SRS also has two more advantages over the commonly used ReLU function: (i) it has nonzero derivative in the negative portion of the function, and (ii) bounded output, i.e., the function takes values in the range $\left[\frac{\alpha \beta }{\beta -\alpha e}\right.,\alpha )$, which is in turn controlled by the α and β parameters

We used two different versions of this activation, depending on whether the parameter β is fixed (labeled here as Soft Learnable) or not (labeled here as Soft Learnable2).

#### 3.4.7. Splash

Splash [64] is another modification of APLU that makes the function symmetric. In the definition of APLU, let $a_{i}$ and $b_{i}$ be the learnable parameters leading to $APLU_{a_{i},b_{i}}\left(x\right)$. Then, Splash is defined as

(29) $$Splash_{a_{i}^{+},a_{i}^{-},b_{i}}\left(x\right)=APLU_{a_{i}^{+},b_{i}}\left(x\right)+APLU_{a_{i}^{-},b_{i}}\left(-x\right).$$

This equation’s hinges are symmetric with respect to the origin. The authors in [65] claim that this network is more robust against adversarial attacks.

#### 3.4.8. 2D MeLU (New)

The 2D Mexican ReLU (2D MeLU) is a novel activation function presented here that is not defined component-wise; instead, every output neuron depends on two input neurons. If a layer has N neurons (or channels), its output is defined as

(30) $$y_{i}=PReLU^{c_{0}}\left(x_{i}\right)+PReLU^{c_{0}}\left(x_{i+1}\right)+\sum_{u,v=1}^{k-1}c_{j}\;\phi^{a_{u,v},\lambda_{\max\left(u,v\right)}}\left(x_{i,}x_{i+1}\right),$$

where $\phi^{a_{j},\lambda_{j}}\left(x_{i,}x_{i+1}\right)=\max\left(\lambda_{j}-\left|\left(x_{i},x_{i+1}\right)-a_{u,v}\right|,0\right)$.

The parameter $a_{u,v}$ is a two-dimensional vector whose entries are the same as those used in MeLU. In other words, $a_{u,v}=\left(a_{u},a_{v}\right)$ as defined in Table 1. Likewise, $\lambda_{\max\left(u,v\right)}$ is defined as it is for MeLU in Table 1.

#### 3.4.9. MeLU + GaLU (New)

MeLU + GaLU is an activation function presented here that is, as its name suggests, the weighted sum of MeLU and GaLU:

(31) $$y_{i}=\left(1-a_{i}\right)MeLU\left(x_{i}\right)+a_{i}\;\mathrm{GaLU}\left(x_{i}\right),\;$$

where $a_{i}$ is a learnable parameter.

#### 3.4.10. Symmetric MeLU (New)

Symmetric MeLU is the equivalent of MeLU, but it is symmetric like Splash. Symmetric MeLU is defined as

(32) $$y_{i}=MeLU\left(x_{i}\right)+MeLU\left(-x_{i}\right),$$

where the coefficients of the two MeLUs are the same. In other words, the *k* coefficients of $MeLU\left(x_{i}\right)$ are the same as $MeLU\left(-x_{i}\right)$.

#### 3.4.11. Symmetric GaLU (New)

Symmetric GaLU is the equivalent of symmetric MeLU but uses GaLU instead of MeLU. Symmetric GaLU is defined as

(33) $$y_{i}=GaLU\left(x_{i}\right)+GaLU\left(-x_{i}\right),$$

where the coefficients of the two GaLUs are the same. In other words, the *k* coefficients of $GaLU\left(x_{i}\right)$ are the same as $GaLU\left(-x_{i}\right).$

#### 3.4.12. Flexible MeLU (New)

Flexible MeLU is a modification of MeLU where the peaks of the Mexican function are also learnable. This feature makes it more similar to APLU since its points of nondifferentiability are also learnable. Compared to MeLU, APLU has more hyperparameters.

## 4. Building CNN Ensembles

One of the objectives of this study is to use several methods for combining the two CNNs with the different activation functions discussed above. Two methods are in need of discussion: Sequential Forward Floating Selection (SFFS) [71] and the stochastic method for combining CNNs introduced in [47].

### 4.1. Sequential Forward Floating Selection (SFFS)

A popular method for selecting an optimal set of descriptors, SFFS [71], has been adapted for selecting the best performing/independent classifiers to be added to the ensemble. In applying the SFFS method, each model to be included in the final ensemble is selected by adding, at each step, the model which provides the highest increment in performance compared to the existing subset of models. Then, a backtracking step is performed to exclude the worst model from the actual ensemble.

This method for combining CNNs is labeled Selection in the experimental section. Since SFFS requires a training phase, we perform a leave-one-out data set selection to select the best-suited models.

### 4.2. Stochastic Method (Stoc)

The stochastic approach [47] involves randomly substituting all the activations in a CNN architecture with a new one selected from a pool of potential candidates. Random selection is repeated many times to generate a set of networks that will be fused together. The candidate activation functions within a pool differ depending on the CNN architecture. Some activation functions appear to perform poorly and some quite well on a given CNN, with quite a large variance. The activation functions included in the pools for each of the CNNs tested here are provided in Table 3. The CNN ensembles randomly built from these pools varied in size, as is noted in the experimental section, which investigates the different ensembles. Ensemble decisions are combined by sum rule, where the softmax probabilities of a sample given by all the networks are averaged, and the new score is used for classification. The stochastic method of combining CNNs is labeled Stoc in the experimental section.

It should be noted that there is no risk of overfitting in the proposed ensemble. The replacement is randomly performed; we did not choose any ad hoc data sets. Overfitting could occur if we chose the Activation Functions (AFs) ad hoc data sets. The aim of this work is to propose an ensemble based on stochastic selection of AFs in order to avoid any risk of overfitting. The disadvantage of our approach is the increased computation time needed to generate the ensembles. As a final note, since 2D MeLU, Splash, and SRS obtain low performance when run with MI = 255 using VGG16, we ran those tests on only a few data sets; those AFs that demonstrate poor performance were cut to reduce computational time.

## 5. Experimental Results

### 5.1. Biomedical Data Sets

There are no fixed definitions of small/midsize data sets that would apply to all fields in data mining. Whether a data set is considered large or small is relative to the task and the publication date of the research. As many deep learning algorithms require large data sets to avoid overfitting, the expectation today is to produce extremely large data sets. We claim that if the data set contains fewer than 1000 images, then it is small, and if the number of images is between 1001 and 10,000, we say that it is midsize.

Each of the activation functions detailed in Section 3 is tested on the CNNs using the following fifteen publicly available biomedical data sets:

1. CH (CHO data set [72]): this is a data set containing 327 fluorescence microscopy images of Chinese hamster ovary cells divided into five classes: antigiantin, Hoechst 33,258 (DNA), antilamp2, antinop4, and antitubulin.
2. HE (2D HeLa data set [72]): this is a balanced data set containing 862 fluorescence microscopy images of HeLa cells stained with various organelle-specific fluorescent dyes. The images are divided into ten classes of organelles: DNA (Nuclei); ER (Endoplasmic reticulum); Giantin, (cis/medial Golgi); GPP130 (cis Golgi); Lamp2 (Lysosomes); Nucleolin (Nucleoli); Actin, TfR (Endosomes); Mitochondria; and Tubulin.
3. RN (RNAi data set [73]): this is a data set of 200 fluorescence microscopy images of fly cells (D. melanogaster) divided into ten classes. Each class contains 1024 × 1024 TIFF images of phenotypes produced from one of ten knock-down genes, the IDs of which form the class labels.
4. MA (C. elegans Muscle Age data set [73]): this data set is for classifying the age of a nematode given twenty-five images of C. elegans muscles collected at four ages representing the classes.
5. TB (Terminal Bulb Aging data set [73]): this is the companion data set to MA and contains 970 images of C. elegans terminal bulbs collected at seven ages representing the classes.
6. LY (Lymphoma data set [73]): this data set contains 375 images of malignant lymphoma representative of three types: Chronic Lymphocytic Leukemia (CLL), Follicular Lymphoma (FL), and Mantle Cell Lymphoma (MCL).
7. LG (Liver Gender Caloric Restriction (CR) data set [73]): this data set contains 265 images of liver tissue sections from six-month-old male and female mice on a CR diet; the two classes represent the gender of the mice.
8. LA (Liver Aging Ad libitum data set [73]): this data set contains 529 images of liver tissue sections from female mice on an ad libitum diet divided into four classes representing the age of the mice.
9. CO (Colorectal Cancer [74]): this is a Zenodo data set (record: 53169#.WaXjW8hJaUm) of 5000 histological images (150 x 150 pixels each) of human colorectal cancer divided into eight classes.
10. BGR (Breast Grading Carcinoma [75]): this is a Zenodo data set (record: 834910#.Wp1bQ-jOWUl) that contains 300 annotated histological images of twenty-one patients with invasive ductal carcinoma of the breast representing three classes/grades 1–3.
11. LAR (Laryngeal data set [76]): this is a Zenodo data set (record: 1003200#.WdeQcnBx0nQ) containing 1320 images of thirty-three healthy and early-stage cancerous laryngeal tissues representative of four tissue classes.
12. HP (set of immunohistochemistry images from the Human Protein Atlas [77]): this is a Zenodo data set (record: 3875786#.XthkoDozY2w) of 353 images of fourteen proteins in nine normal reproductive tissues belonging to seven subcellular locations. The data set in [77] is partitioned into two folds, one for training (177 images) and one for testing (176 images).
13. RT (2D 3T3 Randomly CD-Tagged Images: Set 3 [78]): this collection of 304 2D 3T3 randomly CD-tagged images was created by randomly generating CD-tagged cell clones and imaging them by automated microscopy. The images are divided into ten classes. As in [78], the proteins are put into ten folds so that images in the training and testing sets never come from the same protein.
14. LO (Locate Endogenous data set [79]): this fairly balanced data set contains 502 images of endogenous cells divided into ten classes: Actin-cytoskeleton, Endosomes, ER, Golgi, Lysosomes, Microtubule, Mitochondria, Nucleus, Peroxisomes, and PM. This data set is archived at https://integbio.jp/dbcatalog/en/record/nbdc00296 (accessed on 9 August 2022).
15. TR (Locate Transfected data [79]): this is a companion data set to LO. TR contains 553 images divided into the set same ten classes as LO but with the additional class of Cytoplasm for a total of eleven classes.

Data sets 1–8 can be downloaded at https://ome.grc.nia.nih.gov/iicbu2008/ (accessed on 9 August 2022), data sets 9–12 can be found on Zenodo at https://zenodo.org/record/ (accessed on 9 August 2022) by concatenating the data set’s Zenodo record number provided in the descriptions above to this URL. Data set 13 is available at http://murphylab.web.cmu.edu/data/#RandTagAL (accessed on 9 August 2022), and data sets 14 and 15 are available on request. Unless otherwise noted, the five-fold cross-validation protocol is applied (see Table 3 for details), and the Wilcoxon signed-rank test [80] is the measure used to validate experiments.

### 5.2. Experimental Results

Reported in Table 4 and Table 5 is the performance (accuracy) of the different activation functions on the CNN topologies VGG16 and ResNet50, each trained with a batch size (BS) of 30 and a learning rate (LR) of 0.0001 for 20 epochs (the last fully connected layer has an LR 20 times larger than the rest of the layers (i.e., 0.002)), except the stochastic architectures that are trained for 30 epochs (because of slower convergence). The reason for selecting these settings was to reduce computation time. Images were augmented with random reflections on both axes and two independent random rescales of both axes by two factors uniformly sampled in [1,2] (using MATLAB data augmentation procedures). The objective was to rescale both the vertical and horizontal proportions of the new image. For each stochastic approach, a set of 15 networks was built and combined by sum rule. We trained the models using MATLAB 2019b; however, the pre-trained architectures of newer versions perform better.

The performance (accuracy) of the following ensembles is reported in Table 4 and Table 5:

- ENS: sum rule of {MeLU (k=8), Leaky ReLU, ELU, MeLU (k=4), PReLU, SReLU, APLU, ReLU} (if maxInput=1) or {MeLU (k=8), MeLU (k=4), SReLU, APLU, ReLU} (if maxInput=255);
- eENS: sum rule of the methods that belong to ENS considering both maxInput=1 and maxInput=255;
- ENS_G: as in ENS but Small GaLU and GaLU are added, and in both cases maxInput=1 or maxInput=255;
- eENS_G: sum rule of the methods that belong to ENS_G but considering maxInput=1 and maxInput=255;
- ALL: sum rule among all the methods reported in Table 4 with maxInput=1 or maxInput=255. Notice that when the methods with maxInput=255 are combined, standard ReLU is also added to the fusion. Due to computation time, some activation functions are not combined with VGG16 and so are not considered;
- eALL: sum rule among all the methods, both with maxInput=1 and maxInput=255. Due to computation time, some activation functions are not combined with VGG16 and thus are not considered in an ensemble;
- 15ReLU: ensemble obtained by the fusion of 15 ReLU models. Each network is different because of the stochasticity of the training process;
- Selection: ensemble selected using SFFS (see Section 3.1);
- Stoc_1: MeLU(k=8), Leaky ReLU, ELU, MeLU(k=4), PReLU, SReLU, APLU, GaLU, sGaLU. A maxInput=255 has been used in the stochastic approach (see Section 3.2);
- Stoc_2: the same nine functions of Stoc_1 and an additional set of seven activation functions: ReLU, Soft Learnable, PDeLU, learnableMish, SRS, Swish Learnable, and Swish. A maxInput=255 has been used;
- Stoc_3: same as Stoc_2 but excluding all the activation functions proposed in [46,47,61] (i.e., MeLU, GaLU, and sGaLU);
- Stoc_4: the ensemble detailed in Section 4.

The most relevant results reported in Table 4 on ResNet50 can be summarized as follows:

- ensemble methods outperform stand-alone networks. This result confirms previous research showing that changing activation functions is a viable method for creating ensembles of networks. Note how well 15ReLU outperforms (*p*-value of 0.01) the stand-alone ReLU;
- among the stand-alone ResNet50 networks, ReLU is not the best activation function. The two activations that reach the highest performance on ResNet50 are MeLU (k=8) with maxInput=255 and Splash with maxInput=255. According to the Wilcoxon signed rank test, MeLU (k=8) with maxInput=255 outperforms ReLU with a *p*-value of 0.1. There is no statistical difference between MeLU (k=8) and Splash (with maxInput=255 for both);
- according to the Wilcoxon signed rank test, Stoc_4 and Stoc_2 are similar in performance, and both outperform the other stochastic approach with a *p*-value of 0.1;
- Stoc_4 outperforms eALL, 15ReLU, and Selection with a *p*-value of 0.1. Selection outperforms 15ReLU with *p*-value of 0.01, but Selection’s performance is similar to eALL.

**Figure 5:**
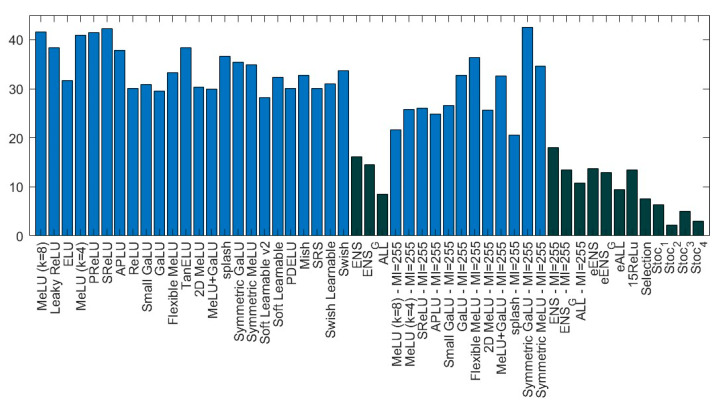
Average rank (lower is better) obtained by different AFs and ensembles coupled with ResNet50 (light blue represents stand-alone methods and dark blue, ensembles).

Examining Figure 5, which illustrates the average rank of the different methods used in Table 4, with ensembles in dark blue and stand-alone in light blue, it is clear that:

(a) there is not a clear winner among the different AFs;
(b) ensembles work better with respect to stand-alone approaches;
(c) the methods named Sto_x work better with respect to other ensembles.

The most relevant results reported in Table 5 on VGG16 can be summarized as follows:

- again, the ensemble methods outperform the stand-alone CNNs. As was the case with ResNet50, 15ReLU strongly outperforms (*p*-value of 0.01) the stand-alone CNNs with ReLU;
- among the stand-alone VGG16 networks, ReLU is not the best activation function. The two activations that reach the highest performance on V6616 are MeLU (k=4) with maxInput=255 and GaLU with maxInput=255. According to the Wilcoxon signed rank test, there is no statistical difference between ReLU, MeLU (k=4), MI=255, and GaLU, MI =255;
- interestingly, ALL with maxInput= 1 outperforms eALL with *p*-value of 0.05;
- Stoc_4 outperforms 15ReLU with *p*-value of 0.01, but the performance of Stoc_4 is similar to eALL, ALL (maxInput=1), and Selection.

Considering both ResNet50 and Vgg16, the best AF is MeLU (*k* = 8), MI = 255. It outperforms ReLU with a *p*-value 0.1 in ResNet and a *p*-value of 0.16 in VGG16. Interestingly, the best average AF is a learnable one that works even on small/midsize data sets.

**Figure 6:**
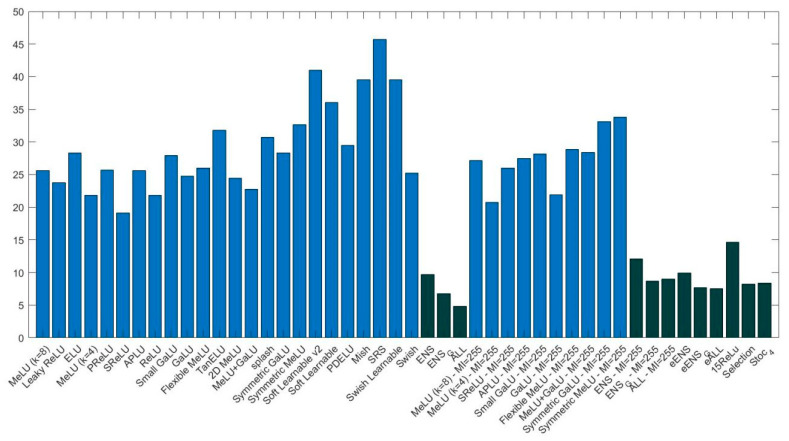
Average rank (lower is better) obtained by different AFs and ensembles coupled with VGG16 (light blue represents stand-alone methods and dark blue, ensembles).

Figure 6 provides a graph reporting the average rank of different AFs and ensembles for VGG16. As with ResNet50 (see Figure 5), it is clear that ensembles of AFs outperform the baseline 15ReLU and stand-alone networks. With VGG16, the performance of Stoc_4 is similar to eALL and Selection.

To further substantiate the power of different AFs in ensembles with small to midsize data sets, in Table 6, we show a further batch of tests comparing 15ReLU and the ensembles built by varying the activation functions to the performance of five different CNN topologies, each trained with a batch size (BS) of 30 and a learning rate (LR) of 0.001 for 20 epochs (the last fully connected layer has an LR 20 times larger than the rest of the layers (i.e., 0.02)) using Matlab2021b. Note that these parameters are slightly different from those of the previous tests. We did not run tests using VGG16 due to computational issues.

The tested CNNs are the following:

- EfficientNetB0 [81]: this CNN does not have ReLU layers, so we only compare the stand-alone CNN with the ensemble labeled 15Reit (15 reiterations of the training).
- MobileNetV2 [82].
- DarkNet53, [83]: this deep network uses LeakyReLU with no ReLU layers; the fusion of 15 standard DarkNet53 models is labeled 15Leaky.
- DenseNet201 [84].
- ResNet50.

As in the previous tests, training images were augmented with random reflections on both axes and two independent random rescales of both axes by two factors uniformly sampled in [1,2] (using MATLAB data augmentation procedures). The objective was to rescale both the vertical and horizontal proportions of the new image.

The most relevant results reported in Table 6 can be summarized as follows:

- the ensembles strongly outperform (*p*-value 0.01) the stand-alone CNN in each topology;
- in MobileNetV2, DenseNet201, and ResNet50, Stoc_4 outperforms 15ReLU (*p*-value 0.05);
- DarkNet53 behaved differently: on this network, 15Leaky and Stoc_4 obtained similar performance.

In Table 7, we report the performance on a few data sets obtained by ResNet50, choosing the optimal values of BS and LR for ReLU. Even with BS and LR optimized for ReLU, the performance of Sto_4 is higher than that obtained by ReLU and 15ReLU.

In Table 8, we report some computation time tests.

The hardware improvements reduce the inference time; there are several applications where it is not a problem to classify 100 images in just a few seconds.

In Table 9, we report the four best AFs for each topology with both MI = 1 and MI = 255.

If we consider the two larger data sets, CO and LAR, the best AF is always a learnable one:

- CO—ResNet: the best is Swish Learnable;
- LAR—ResNet: the best is 2D MeLU;
- CO—VGG16: the best is MeLU + GaLU;
- LAR—VGG16: the best is MeLU (*k* = 4).

It is clear that some of the best AFs are proposed here.

## 6. Conclusions

The goal of this study was to evaluate some state-of-the-art deep learning techniques on medical images and data. Towards this aim, we evaluated the performance of CNN ensembles created by replacing the ReLU layers with activations from a large set of activation functions, including six new activation functions introduced here for the first time (2D Mexican ReLU, TanELU, MeLU + GaLU, Symmetric MeLU, Symmetric GaLU, and Flexible MeLU). Tests were run on two different networks: VGG16 and ResNet50, across fifteen challenging image data sets representing various tasks. Several methods for making ensembles were also explored.

Experiments demonstrate that an ensemble of multiple CNNs that differ only in their activation functions outperforms the results of single CNNs. Experiments also show that, among the single architectures, there is no clear winner.

More studies need to investigate the performance gains offered by our approach on even more data sets. It would be of value, for instance, to examine whether the boosts in performance our system achieved on the type of data tested in this work would transfer to other types of medical data, such as Computer Tomography (CT) and Magnetic Resonance Imaging (MRI), as well as image/tumor segmentation. Studies such as the one presented here are difficult, however, because investigating CNNs requires enormous computational resources. Nonetheless, such studies are necessary to increase the capacity of deep learners to classify medical images and data accurately.

## Figures and Tables

**Table 1:** Fixed parameters of MeLU with maxInput=256 (these are the same values as in [61]).

J	1	2	3	4	5	6	7
$a_{j}$	512	256	768	128	384	640	896
$\lambda_{j}$	512	256	256	128	128	128	128

**Table 2:** Comparison of the fixed parameters of GaLU and MeLU with maxInput=1.

	J	1	2	3	4	5	6	7
MELU	$a_{j}$	2.00	1.00	3.00	0.50	1.50	2.50	3.50
	$\lambda_{j}$	2.00	1.00	1.00	0.50	0.50	0.50	0.50
GALU	$a_{j}$	1.00	0.50	2.50	0.25	1.25	2.25	3.25
	$\lambda_{j}$	1.00	0.50	0.50	0.25	0.25	0.25	0.25

**Table 3:** Description of the data sets: xCV means a x fold cross-validation; Tr-Te means that training and test set are split by the authors of that data set.

Short Name	Full Name	#Classes	#Samples	Protocol	Image Type
CH	CHO	5	327	5CV	hamster ovary cells
HE	2D HeLa	10	862	5CV	subcellular location
RN	RNAi data set		200	5CV	fly cells
MA	Muscle aging	4	237	5CV	muscles
TB	Terminal Bulb Aging	7	970	5CV	terminal bulbs
LY	Lymphoma	3	375	5CV	malignant lymphoma
LG	Liver Gender	2	265	5CV	liver tissue
LA	Liver Aging	4	529	5CV	liver tissue
CO	Colorectal Cancer	8	5000	10CV	histological images
BGR	Breast grading carcinoma	3	300	5CV	histological images
LAR	Laryngeal data set	4	1320	Tr-Te	laryngeal tissues
HP	Immunohistochemistry images from the human protein atlas	7	353	Tr-Te	reproductive tissues
RT	2D 3T3 Randomly CD-Tagged Cell Clones	10	304	10CV	CD-tagged cell clones
LO	Locate Endogenous	10	502	5CV	subcellular location
TR	Locate Transfected	11	553	5CV	subcellular location

**Table 4:** Performance (accuracy) of activation function obtained using ResNet50.

	Activation	CH	HE	LO	TR	RN	TB	LY	MA	LG	LA	CO	BG	LAR	RT	HP	Avg
ResNet50 MaxInput = 1	MeLU (*k* = 8)	92.92	86.40	91.80	82.91	25.50	56.29	67.47	76.25	91.00	82.48	94.82	89.67	88.79	68.36	48.86	76.23
	Leaky ReLU	89.23	87.09	92.80	84.18	34.00	57.11	70.93	79.17	93.67	82.48	95.66	90.33	87.27	69.72	45.45	77.27
	ELU	90.15	86.74	94.00	85.82	48.00	60.82	65.33	85.00	96.00	90.10	95.14	89.33	89.92	73.50	40.91	79.38
	MeLU (*k* = 4)	91.08	85.35	92.80	84.91	27.50	55.36	68.53	77.08	90.00	79.43	95.34	89.33	87.20	72.24	51.14	76.48
	PReLU	92.00	85.35	91.40	81.64	33.50	57.11	68.80	76.25	88.33	82.10	95.68	88.67	89.55	71.20	44.89	76.43
	SReLU	91.38	85.58	92.60	83.27	30.00	55.88	69.33	75.00	88.00	82.10	95.66	89.00	89.47	69.98	42.61	75.99
	APLU	92.31	87.09	93.20	80.91	25.00	54.12	67.20	76.67	93.00	82.67	95.46	90.33	88.86	71.65	48.30	76.45
	ReLU	93.54	89.88	95.60	90.00	55.00	58.45	77.87	90.00	93.00	85.14	94.92	88.67	87.05	69.77	48.86	81.18
	Small GaLU	92.31	87.91	93.20	91.09	52.00	60.00	72.53	90.00	95.33	87.43	95.38	87.67	88.79	67.57	44.32	80.36
	GaLU	92.92	88.37	92.20	90.36	41.50	57.84	73.60	89.17	92.67	88.76	94.90	90.33	90.00	72.98	48.86	80.29
	Flexible MeLU	91.69	88.49	93.00	91.64	38.50	60.31	73.33	88.33	95.67	87.62	94.72	89.67	86.67	67.35	44.32	79.42
	TanELU	93.54	86.16	90.60	90.91	40.00	58.56	69.60	86.25	95.33	83.05	94.80	87.67	86.89	73.95	43.18	78.69
	2D MeLU	91.69	87.67	93.00	91.64	48.00	60.41	72.00	91.67	96.00	88.38	95.42	89.00	87.58	70.53	42.61	80.37
	MeLU + GaLU	93.23	88.02	93.40	92.91	54.50	59.18	72.53	89.58	95.33	86.29	95.34	88.64	88.64	69.29	43.18	80.67
	Splash	93.54	87.56	93.80	90.00	47.50	55.98	72.00	82.92	94.33	84.19	95.02	86.00	87.12	75.70	42.61	79.21
	Symmetric GaLU	93.85	84.19	92.80	89.45	47.50	58.66	72.80	87.08	95.33	82.67	94.44	87.33	87.80	71.52	52.84	79.88
	Symmetric MeLU	92.62	86.63	92.40	89.27	50.00	60.62	72.27	85.42	95.00	85.14	94.72	90.00	87.58	66.71	50.57	79.93
	Soft Learnable v2	93.93	87.33	93.60	92.55	46.00	60.31	69.07	89.58	94.67	86.10	95.00	89.67	87.05	73.72	54.55	80.87
	Soft Learnable	94.15	87.44	93.40	90.36	47.00	59.18	67.73	88.33	95.00	85.52	95.52	89.33	88.26	72.04	46.59	79.99
	PDELU	94.15	87.21	92.00	91.64	51.50	56.70	70.93	89.58	96.33	86.67	95.08	89.67	88.18	72.76	46.59	80.59
	Mish	95.08	87.56	93.20	91.82	45.00	58.45	69.07	86.67	95.33	86.67	95.48	90.00	88.41	53.41	34.09	78.01
	SRS	93.23	88.84	93.40	91.09	51.50	60.10	69.87	88.75	95.00	86.48	95.72	88.33	89.47	54.06	48.86	79.64
	Swish Learnable	93.54	87.91	94.40	91.64	48.00	59.28	69.33	88.75	95.33	83.24	96.10	90.00	89.32	41.15	39.77	77.85
	Swish	94.15	88.02	94.20	90.73	48.50	59.90	70.13	89.17	92.67	86.10	95.66	87.67	87.65	65.05	32.39	78.79
	ENS	95.38	89.53	97.00	89.82	59.00	62.78	76.53	86.67	96.00	91.43	96.60	91.00	89.92	74.00	50.00	83.04
	ENS_G	93.54	90.70	97.20	92.73	56.00	63.92	77.60	90.83	96.33	91.43	96.42	90.00	90.00	73.76	50.00	83.36
	ALL	97.23	91.16	97.20	95.27	58.00	65.15	76.80	92.92	98.00	90.10	96.58	90.00	90.38	74.67	53.98	84.49
ResNet50 MaxInput = 255	MeLU (*k* = 8)	94.46	89.30	94.20	92.18	54.00	61.86	75.73	89.17	97.00	88.57	95.60	87.67	88.71	72.09	52.27	82.18
	MeLU (*k* = 4)	92.92	90.23	95.00	91.82	57.00	59.79	78.40	87.50	97.33	85.14	95.72	89.33	88.26	66.20	48.30	81.52
	SReLU	92.31	89.42	93.00	90.73	56.50	59.69	73.33	91.67	98.33	88.95	95.52	89.67	87.88	68.94	48.30	81.61
	APLU	95.08	89.19	93.60	90.73	47.50	56.91	75.20	89.17	97.33	87.05	95.68	89.67	89.47	71.44	51.14	81.27
	Small GaLU	93.54	87.79	95.60	89.82	55.00	63.09	76.00	90.42	95.00	85.33	95.08	89.67	89.77	72.14	45.45	81.58
	GaLU	92.92	87.21	92.00	91.27	47.50	60.10	74.13	87.92	96.00	86.86	95.56	89.33	87.73	70.26	44.32	80.20
	Flexible MeLU	92.62	87.09	91.60	91.09	48.50	57.01	69.60	86.67	95.00	87.81	95.26	89.00	88.11	70.83	46.59	79.78
	2D MeLU	95.08	90.23	93.00	91.45	54.00	57.42	69.60	90.42	96.00	87.43	91.84	87.67	90.76	73.44	54.55	81.52
	MeLU + GaLU	93.23	87.33	92.20	90.91	54.00	58.66	73.87	89.58	95.33	88.76	95.42	86.33	86.74	70.91	48.86	80.92
	Splash	96.00	87.67	92.80	93.82	50.50	60.62	78.13	89.58	96.67	87.81	95.18	90.33	91.36	68.81	51.70	82.06
	Symmetric GaLU	92.00	85.58	91.20	89.64	43.50	57.94	70.93	79.58	91.33	85.14	95.34	87.33	85.98	69.37	47.16	78.13
	Symmetric MeLU	92.92	88.37	93.40	92.00	44.00	58.56	69.60	91.67	93.33	84.00	94.94	87.33	88.79	70.30	44.89	79.60
	ENS	93.85	91.28	96.20	93.27	59.00	63.30	77.60	91.67	98.00	87.43	96.30	89.00	89.17	71.11	50.00	83.14
	ENS_G	95.08	91.28	96.20	94.18	63.00	64.85	78.67	92.50	97.67	87.62	96.54	89.67	89.77	71.36	51.14	83.96
	ALL	96.00	91.16	96.60	94.55	60.50	64.74	77.60	92.92	97.67	89.52	96.62	89.33	90.68	74.37	52.27	84.30
eENS		94.77	91.40	97.00	92.91	60.00	64.74	77.87	88.75	98.00	90.10	96.50	90.00	89.77	73.23	50.57	83.70
eENS_G		95.08	91.28	96.80	93.45	62.50	65.26	78.93	91.67	96.67	90.48	96.60	89.33	89.85	73.60	50.00	84.10
eALL		96.92	91.28	97.20	95.45	60.50	64.64	77.87	93.75	97.67	90.10	96.58	89.67	90.68	74.37	52.27	84.59
15ReLU		95.40	91.10	96.20	95.01	58.50	64.80	76.00	92.90	97.30	89.30	96.30	90.00	90.04	73.00	50.57	83.76
Selection		96.62	91.40	97.00	95.09	60.00	64.85	77.87	93.75	98.00	90.29	96.78	90.00	90.98	74.04	54.55	84.74
Stoc_1		97.81	91.51	96.66	95.87	60.04	65.83	80.02	92.96	99.09	91.24	96.61	90.77	91.03	74.20	50.57	84.95
Stoc_2		98.82	93.42	97.87	96.48	65.58	66.92	85.65	92.94	99.77	94.33	96.63	91.36	92.34	76.83	54.55	86.89
Stoc_3		99.43	93.93	98.04	96.06	64.55	66.41	83.24	90.04	96.04	93.93	96.72	92.05	91.34	75.89	51.70	85.95
Stoc_4		98.77	92.09	97.40	96.55	63.00	67.01	81.87	93.33	100	93.52	96.72	93.00	92.27	76.38	51.70	86.24

**Table 5:** Activation performance (accuracy) on VGG16.

	ACTIVATION	CH	HE	LO	TR	RN	TB	LY	MA	LG	LA	CO	BG	LAR	RT	HP	AVG
VGG16 MAXINPUT = 1	MeLU (*k* = 8)	99.69	92.09	98.00	92.91	59.00	60.93	78.67	87.92	86.67	93.14	95.20	89.67	90.53	73.73	42.61	82.71
	Leaky ReLU	99.08	91.98	98.00	93.45	66.50	61.13	80.00	92.08	86.67	91.81	95.62	91.33	88.94	74.86	38.07	83.30
	ELU	98.77	93.95	97.00	92.36	56.00	59.69	81.60	90.83	78.33	85.90	95.78	93.00	90.45	71.55	40.91	81.74
	MeLU (*k* = 4)	99.38	91.16	97.60	92.73	64.50	62.37	81.07	89.58	86.00	89.71	95.82	89.67	93.18	75.20	42.61	83.37
	PReLU	99.08	90.47	97.80	94.55	64.00	60.00	81.33	92.92	78.33	91.05	95.80	92.67	90.38	73.74	35.23	82.49
	SReLU	99.08	91.16	97.00	93.64	65.50	60.62	82.67	90.00	79.33	93.33	96.10	94.00	92.58	76.80	45.45	83.81
	APLU	99.08	92.33	97.60	91.82	63.50	62.27	77.33	90.00	82.00	92.38	96.00	91.33	90.98	76.58	34.66	82.52
	ReLU	99.69	93.60	98.20	93.27	69.50	61.44	80.80	85.00	85.33	88.57	95.50	93.00	91.44	73.68	40.34	83.29
	Small GaLU	98.46	91.63	97.80	91.35	64.50	59.79	80.53	89.58	77.33	92.76	95.70	91.67	91.97	72.63	44.32	82.66
	GaLU	98.46	94.07	97.40	92.36	65.00	59.07	81.07	92.08	75.67	93.71	95.68	88.67	91.74	75.81	39.20	82.66
	Flexible MeLU	97.54	94.19	96.60	94.91	59.00	62.68	77.07	90.00	89.00	91.81	95.94	92.67	89.92	72.15	38.64	82.80
	TanELU	97.85	93.14	97.00	92.36	61.00	61.44	72.80	89.17	77.33	91.62	95.28	89.67	90.23	72.84	43.75	81.69
	2D MeLU	97.85	93.72	97.20	92.73	61.00	61.34	81.60	91.25	92.33	94.48	95.86	89.67	92.35	71.91	38.64	83.46
	MeLU + GaLU	98.15	93.72	98.20	93.64	60.00	60.82	77.60	92.08	81.00	93.14	95.54	92.33	89.47	75.60	47.16	83.23
	Splash	97.85	92.79	97.80	92.18	58.50	62.06	75.73	88.33	83.67	85.90	95.02	91.67	90.15	74.29	42.05	81.86
	Symmetric GaLU	99.08	92.79	97.20	92.91	60.50	60.00	78.93	88.33	79.33	91.62	95.52	92.67	91.67	73.91	40.34	82.32
	Symmetric MeLU	98.46	92.91	96.60	92.18	56.50	59.69	74.93	90.00	85.00	87.05	94.76	90.33	90.68	72.87	41.48	81.56
	Soft Learnable v2	95.69	87.91	94.60	93.45	34.50	55.57	50.67	77.50	64.67	29.71	94.08	67.67	92.35	68.96	35.80	69.54
	Soft Learnable	98.15	92.91	97.00	91.82	47.50	54.33	62.13	86.67	95.67	65.90	95.04	84.33	90.38	71.08	40.34	78.21
	PDELU	98.77	93.60	96.40	92.18	59.00	58.25	76.80	87.92	87.67	89.33	95.36	90.33	91.74	75.24	42.05	82.30
	Mish	96.31	90.70	94.60	93.64	18.50	46.80	54.13	66.67	73.67	56.38	93.88	80.00	82.73	73.89	44.32	71.08
	SRS	71.08	59.19	45.00	51.64	29.50	31.44	57.60	61.25	61.00	45.33	86.88	57.00	67.50	39.74	19.32	52.23
	Swish Learnable	97.54	91.86	97.00	93.64	43.50	54.64	66.67	87.08	81.00	79.43	94.46	81.00	85.23	70.02	35.23	77.22
	Swish	98.77	92.56	96.80	93.64	63.50	58.97	80.80	90.00	89.00	93.14	94.68	93.33	91.74	75.24	39.77	83.46
	ENS	99.38	93.84	98.40	95.64	68.00	65.67	85.07	92.08	85.00	96.38	96.74	94.33	92.65	75.55	44.89	85.57
	ENS_G	99.69	94.65	99.00	95.45	72.00	64.95	86.93	92.50	83.33	97.14	96.72	94.67	92.65	75.56	45.45	86.07
	ALL	99.69	95.35	98.80	95.45	72.00	66.80	84.00	94.17	85.67	97.14	96.66	95.00	93.18	75.85	48.30	86.53
VGG16 MAXINPUT = 255	MeLU (*k* = 8)	99.69	92.09	97.40	93.09	59.50	60.82	80.53	88.75	80.33	88.57	95.94	90.33	88.33	73.01	47.73	82.40
	MeLU (*k* = 4)	99.38	91.98	98.60	92.55	66.50	59.59	84.53	91.67	88.00	94.86	95.46	93.00	93.03	72.21	38.64	84.00
	SReLU	98.77	93.14	97.00	92.18	65.00	62.47	77.60	89.58	76.00	96.00	95.84	94.33	89.85	74.04	42.61	82.96
	APLU	98.77	92.91	97.40	93.09	63.00	57.32	82.67	90.42	77.00	90.67	94.90	93.00	91.21	75.65	36.36	82.29
	Small GaLU	99.38	92.91	97.00	92.73	50.50	62.16	78.40	90.42	73.00	94.48	95.32	92.00	90.98	73.61	42.61	81.70
	GaLU	98.77	92.91	97.60	93.09	66.50	59.48	83.47	90.83	95.00	85.52	95.96	91.67	93.41	75.45	38.64	83.88
	Flexible MeLU	99.08	95.00	97.20	93.45	62.00	55.98	76.80	89.17	83.00	88.57	95.64	91.33	91.29	73.00	37.50	81.93
	MeLU + GaLU	98.46	94.42	96.80	92.00	54.50	60.82	79.73	90.83	78.67	93.33	96.26	89.67	91.14	74.79	40.34	82.11
	Symmetric GaLU	97.85	92.21	97.40	93.64	58.00	58.14	73.87	91.67	79.33	91.43	95.18	90.33	89.55	74.47	34.09	81.14
	Symmetric MeLU	98.46	92.33	96.80	92.18	56.50	61.24	75.47	89.17	82.00	88.00	95.32	92.67	88.86	74.27	38.07	81.42
	ENS	99.38	93.84	98.80	95.27	68.50	64.23	84.53	92.50	81.33	96.57	96.66	95.00	92.20	75.27	43.75	85.18
	ENS_G	99.38	94.88	98.80	95.64	70.50	65.88	85.87	93.75	81.67	96.38	96.70	95.67	92.80	75.26	44.32	85.83
	ALL	99.69	95.47	98.40	95.45	70.00	63.92	83.73	94.17	82.67	96.38	96.60	95.00	92.73	75.78	45.45	85.69
EENS		99.38	94.07	98.80	95.64	69.00	65.88	85.87	93.33	82.67	96.57	96.88	95.33	92.50	74.99	43.18	85.60
EENS_G		99.69	94.65	99.00	95.27	70.50	65.57	86.93	92.92	83.33	97.71	96.82	95.00	92.42	76.09	44.32	86.01
EALL		99.69	95.70	98.80	95.45	71.50	65.98	83.73	94.58	85.67	96.38	96.70	95.00	92.50	75.42	47.16	86.28
15RELU		99.08	95.35	98.60	94.91	64.50	64.64	79.20	95.00	83.00	92.76	96.38	94.00	92.42	74.34	50.57	84.98
SELECTION		99.69	95.26	98.60	94.91	71.00	64.85	86.67	94.58	84.67	95.24	96.72	94.33	93.56	75.48	47.16	86.18
STOC_4		99.69	96.05	98.60	95.27	74.50	67.53	83.47	95.00	84.00	95.62	96.78	92.67	93.48	74.87	51.70	86.61

**Table 6:** Ensemble performance (accuracy) on a set of different topologies (due to the high computational time for CO we have run only 4 Sto_4 Densenet201).

EfficientNetB0	CH	HE	LO	TR	RN	TB	LY	MA	LG	LA	CO	BG	LAR	RT	HP	Avg
ReLU	94.46	91.28	94.80	92.18	68.50	62.58	88.80	92.50	97.33	96.76	95.04	90.67	87.35	71.21	52.27	85.05
15Reit	96.00	92.09	95.40	93.82	74.00	65.98	89.07	93.33	97.00	98.29	95.60	90.00	88.94	71.61	61.36	86.83
**MobileNetV2**	**CH**	**HE**	**LO**	**TR**	**RN**	**TB**	**LY**	**MA**	**LG**	**LA**	**CO**	**BG**	**LAR**	**RT**	**HP**	**Avg**
ReLU	98.15	92.91	97.40	92.91	69.00	64.54	76.00	91.67	96.67	96.76	94.54	89.00	90.23	69.53	50.57	84.65
15ReLU	99.08	95.23	98.80	95.64	75.00	70.41	80.27	95.42	98.00	97.71	95.46	90.67	91.52	69.24	55.11	87.17
Stoc_4	99.08	95.35	99.20	98.36	84.00	76.91	87.20	94.58	100	99.62	95.50	94.00	95.08	77.02	63.64	90.63
**DarkNet53**	**CH**	**HE**	**LO**	**TR**	**RN**	**TB**	**LY**	**MA**	**LG**	**LA**	**CO**	**BG**	**LAR**	**RT**	**HP**	**Avg**
ReLU	98.77	93.60	98.00	95.82	71.00	67.84	81.33	71.25	98.00	96.95	92.02	91.67	91.44	67.12	53.98	84.58
15Leaky	99.69	95.12	99.20	99.45	89.00	77.94	91.73	89.17	100	99.81	95.56	93.00	93.56	76.02	61.93	90.74
Stoc_4	99.69	95.93	98.80	98.80	88.00	77.73	96.00	88.33	100	99.81	95.28	91.00	92.12	74.33	67.05	90.86
**ResNet50**	**CH**	**HE**	**LO**	**TR**	**RN**	**TB**	**LY**	**MA**	**LG**	**LA**	**CO**	**BG**	**LAR**	**RT**	**HP**	**Avg**
ReLU	97.54	94.19	98.40	95.82	74.50	65.15	80.00	92.08	98.00	96.76	96.26	89.67	91.44	77.21	55.68	86.84
15ReLU	99.08	95.70	99.20	97.27	79.00	69.38	84.27	95.42	97.33	98.10	97.00	91.00	93.79	77.15	59.66	88.89
Stoc_4	99.69	95.47	99.20	98.00	85.00	75.26	91.47	95.00	99.00	99.62	97.02	93.00	94.85	75.18	62.50	90.68
**DenseNet201**	**CH**	**HE**	**LO**	**TR**	**RN**	**TB**	**LY**	**MA**	**LG**	**LA**	**CO**	**BG**	**LAR**	**RT**	**HP**	**Avg**
ReLU	98.73	95.29	98.37	96.92	71.40	66.80	82.20	91.31	98.22	98.12	95.88	91.69	93.96	49.92	54.70	85.56
15ReLU	99.38	96.40	98.40	98.55	79.00	71.24	86.40	94.58	99.67	99.24	97.84	95.33	96.14	77.57	61.36	90.07
Stoc_4	99.69	94.88	99.20	99.27	84.00	76.29	93.87	96.67	100	100	97.84	93.00	95.38	77.67	69.89	91.84

**Table 7:** Performance (accuracy) with optimized BS and LR.

ResNet50		CH	HE	MA	LAR
	ReLU	98.15	95.93	95.83	94.77
	15ReLU	99.08	96.28	97.08	95.91
	Sto_4	99.69	96.40	97.50	96.74

**Table 8:** Inference time of a batch size of 100 images.

GPU	Year GPU	Single ResNet50	Ensemble 15 ResNet50
GTX 1080	2016	0.36 s	5.58 s
Titan Xp	2017	0.31 s	4.12 s
Titan RTX	2018	0.22 s	2.71 s
Titan V100	2018	0.20 s	2.42 s

**Table 9:** The four best AFs are reported (TopXr means X-th position in the rank among the AFs).

Topology	MI	Top1r	Top2r	Top3r	Top4r
ResNet50	1	MeLU + GaLU	SRS	PDELU	Soft Learnable v2
ResNet50	255	MeLU (*k* = 8)	Splash	MeLU (*k* = 4)	2D MeLU
VGG16	1	SReLU	MeLU + GaLU	MeLU (*k* = 4)	ReLU
VGG16	255	GaLU	MeLU (*k* = 4)	SReLU	APLU
